# Dr. John Rand

**Published:** 1912-09-28

**Authors:** 


Dr. John Rand, of Dulwich, has died in his seventy-seventh year. He started his medical career as an apprentice to a country practitioner, and then "walked" in Guy's Hospital. After qualifying in 1858 he served as house physician and house surgeon in his own hospital, and also studied in Paris. He first took up country practice in the Eastern Counties, but after a severe illness he joined a firm in Dulwich, having meanwhile obtained the Fellowship of the Royal College of Surgeons of England in 1869. In Dulwich he became both successful and popular, and his retirement was marked by a presentation from his numerous friends and grateful patients. He was twice president of the Sydenham District Medical Society, and did a great deal to start the East Dulwich Provident Dispensary—still one of the most flourishing of these institutions.

